# The English National Cohort Study of Flooding and Health: the change in the prevalence of psychological morbidity at year two

**DOI:** 10.1186/s12889-018-5236-9

**Published:** 2018-03-07

**Authors:** Daiga Jermacane, Thomas David Waite, Charles R. Beck, Angie Bone, Richard Amlôt, Mark Reacher, Sari Kovats, Ben Armstrong, Giovanni Leonardi, G. James Rubin, Isabel Oliver

**Affiliations:** 1 European Programme for Intervention Epidemiology Training (EPIET), European Centre for Disease Prevention and Control, (ECDC), Stockholm, Sweden; 2 National Infection Service, Public Health England, 2, Rivergate, Bristol, BS1 6EH UK; 3 Centre for Radiation, Chemicals and Environmental Hazards, Public Health England, OX11 0RQ Chilton, Didcot, Oxfordshire UK; 4 School of Social and Community Medicine, University of Bristol, Bristol, UK; 5 Emergency Response Department, Public Health England, Porton Down, Wilts, SP4 0JG Salisbury, UK; 6 NIHR Health Protection Research Unit in Environmental Change and Health at the London School of Hygiene and Tropical Medicine, London, UK; 7 NIHR Health Protection Research Unit in Emergency Preparedness and Response at King’s College London, London, UK; 8 NIHR Health Protection Research Unit in Evaluation of Interventions at the University of Bristol, Bristol, UK; 9 National Infection Service, Public Health England, Cambridge, UK; 10 Cambridge Institute of Public Health, Cambridge, UK

## Abstract

**Background:**

The longer term impact of flooding on health is poorly understood. In 2015, following widespread flooding in the UK during winter 2013/14, Public Health England launched the English National Study of Flooding and Health. The study identified a higher prevalence of probable psychological morbidity one year after exposure to flooding. We now report findings after two years.

**Methods:**

In year two (2016), a self-assessment questionnaire including flooding-related exposures and validated instruments to screen for probable anxiety, depression and post-traumatic stress disorder (PTSD) was sent to all participants who consented to further follow-up. Participants exposure status was categorised according to responses in year one; we assessed for exposure to new episodes of flooding and continuing flood-related problems in respondents homes. We calculated the prevalence and odds ratio for each outcome by exposure group relative to unaffected participants, adjusting for confounders. We used the McNemar test to assess change in outcomes between year one and year two.

**Results:**

In year two, 1064 (70%) people responded. The prevalence of probable psychological morbidity remained elevated amongst flooded participants [*n* = 339] (depression 10.6%, anxiety 13.6%, PTSD 24.5%) and disrupted participants [*n* = 512] (depression 4.1%, anxiety 6.4%, PTSD 8.9%), although these rates were reduced compared to year one. A greater reduction in anxiety 7.6% (95% confidence interval [CI] 4.6–9.9) was seen than depression 3.8% (95% CI 1.5–6.1) and PTSD: 6.6% (95% CI 3.9–9.2). Exposure to flooding was associated with a higher odds of anxiety (adjusted odds ratio [aOR] 5.2 95%, 95% CI 1.7–16.3) and depression (aOR 8.7, 95% CI 1.9–39.8) but not PTSD. Exposure to disruption caused by flooding was not significantly associated with probable psychological morbidity. Persistent damage in the home as a consequence of the original flooding event was reported by 119 participants (14%). The odds of probable psychological morbidity amongst flooded participants who reported persistent damage, compared with those who were unaffected, were significantly higher than the same comparison amongst flooded participants who did not report persistent damage.

**Conclusions:**

This study shows a continuance of probable psychological morbidity at least two years following exposure to flooding. Commissioners and providers of health and social care services should be aware that the increased need in populations may be prolonged. Efforts to resolve persistent damage to homes may reduce the risk of probable psychological morbidity.

**Electronic supplementary material:**

The online version of this article (10.1186/s12889-018-5236-9) contains supplementary material, which is available to authorized users.

## Background

Flooding is the most frequent global natural hazard. The incidence and impact of flooding events has been increasing world-wide and this trend is set to continue [1, 2].

In the UK, around 1.8 million people live in properties with an annual risk of flooding greater than 1 in 75. This is expected to increase because of climate change and the pressures of development [3]. The winter of 2015/2016 was the second wettest winter on record and a series of storms (including ‘Desmond’ and ‘Eva’) resulted in heavy and sustained rainfall. 17,600 UK properties were flooded. Economic damage was estimated to be about £1.6 billion [4].

The relationship between flood events, their aftermath and population health and wellbeing is complex, and the mechanisms through which wellbeing is affected remain under-investigated [5–7]. Although the level of exposure to floods has been associated with probable psychological morbidity, the paucity of longitudinal studies and unrecognised confounding factors precludes strong conclusions [5, 6, 8–10]. Few studies have investigated the medium to long-term impact of flood events on mental health and there are methodological limitations in the evaluation or comparisons of those results [10].

Many people experience distress after disasters. Personal and collective psychosocial resilience are inherent in many communities and most people will recover with this support [8]. However, some studies, have identified an impact on mental health and suggested that it may last months or years after flooding [5, 10–13]. Multiple factors associated with flooding could underlie psychological distress, including fears and actions taken to protect family or belongings, experience of flooding and long-term uncertainties around insurance [14, 15].

The storms of winter 2013–14 in England were exceptional and brought the wettest winter in 250 years with flood warnings (severe flooding, danger to life) issued to 2.5 million properties by the Environment Agency [16]. Following these events, Public Health England, working with National Institute of Health Research Health Protection Research Units, established the English National Cohort Study of Flooding and Health in order to investigate the longer term impact of flooding on mental health and wellbeing to inform the future public health response to flooding events. Waite and colleagues reported a high prevalence of probable psychological morbidity after one year of follow-up amongst flooded participants: depression 20.1%, anxiety 28.3%, PTSD 36.2% and participants who were not directly flooded but whose lives were disrupted by the incident: depression 9.6%, anxiety 10.7% PTSD 15.2%, compared to those unaffected (2015) [5].

In 2016, we contacted the participants of this study again to understand if the adverse impact of flooding on mental health persists after two years. Our objectives in the second year of the study were to:

1. Assess the prevalence of probable psychological morbidity two years after flooding among participants exposed to flooding or disruption from flooding compared to those unaffected;
2. Estimate the change in the prevalence of probable psychological morbidity (anxiety, depression, PTSD) between the second year and the first year of follow up in participants (a) flooded and (b) disrupted by flooding compared to those unaffected;
3. Investigate whether any changes in prevalence are affected by flood-related factors, disruption-related factors or demographic variables.

## Methods

### Study design

This study is a two year follow-up survey of the English National Study of Flooding and Health designed as longitudinal observational open cohort. The participants are a sample of people living in neighbourhoods in the south of England affected by flooding between 1 December 2013 and 31 March 2014 [5].

### Study population

The original cohort consisted of 2126 responded participants, 1406 of which had provided consent to be followed-up and were invited to complete a questionnaire (Additional file 1). The 718(34%) respondents gave no consent for follow-up and they do not remain in an investigation of this study.

Participants were categorised into three groups according to their exposure to flooding as reported in year one; flooded (i.e. entry of water into any liveable room of the home), disrupted (life disrupted by flooding but no entry of water into a liveable room of the home), and unaffected by flooding [5].

### Data collection

A 21-item questionnaire was used. A link to an electronic copy of the questionnaire was sent to participants who had provided an e-mail address (39%) with a paper copy of the same questionnaire sent by post to the rest (61%).

We used validated instruments and the cut-off scores employed in clinical practice to screen for symptoms suggestive of probable mental health outcomes. The instruments included the Patient Health Questionnaire (PHQ-2) for depression, Generalised Anxiety Disorder scale (GAD-2) for anxiety and Post Traumatic Stress Disorder (PTSD) checklist (PCL-6) for PTSD. Cut-off scores were ≥3 for PHQ-2/GAD-2 and ≥14 for PCL-6 as in year one analysis [17, 18].

The questionnaire also collected information on socio-demographic characteristics including age, sex, date of birth, ethnicity, marital status, household composition and tenure, area of residence, education, employment and the presence of any limiting long term illness as well as questions to identify any ongoing damage (“persistent damage”), ability to resume all liveable rooms as normal with flooding and any new exposures to flooding. “Persistent damage” was defined as ongoing flood related problems to the home, caused by the flooding in 2013/14 and included problems with damp in liveable rooms, visible mould in liveable rooms, problems with damp or water in non-liveable rooms (garage, cellar or basement), sewage (drains) backing up and flooding, problems with a septic tank and problems with other utilities (drinking water, gas, oil, electricity, etc.). These were treated as sub-groups of the flooded and disrupted exposure categories.

Information was also collected on secondary stressors (for example, dealing with insurance issues, repairing home, concerns about health, relationship problems, arguments with neighbours) and status of any insurance claims and repair and renew grant applications [19].

### Statistical methods

A multivariable logistic regression model was constructed to calculate odds ratios for each outcome by exposure group relative to unaffected participants, adjusting for those variables considered a priori from previous literature to be possible risk factors and hence as potential confounders: age group, sex, local authority of residence, ethnicity, marital status, education level, employment and local area deprivation score, based on Index of Multiple Deprivation (IMD). We also carried out a Wald test for the difference in outcome odds in those with and without persistent damage.

The following variables were considered a priori from previous literature to be possible modifiers of associations of flooding with our outcomes: housing tenure, previous experience of flooding, having home insurance, submitting an insurance claim, being sole adult occupant of a property. These were entered separately as interaction terms and tested using a Wald statistic.

Confidence intervals for the change in the prevalence proportion (“reduction between year one and year two”) was calculated by using methods for matched pairs [20]. Each pair was composed of two observations for each participant, outcome in year one and in year two.

Conditional regression models were used to explore the statistical significance of changes in prevalence between year one to year two across groups defined by individual characteristics (demographics) or factors related to flooding or disruption, putative predictors of recovery from probable psychological morbidity.

Participants who provided insufficient data to allow exposure categorisation were excluded from analyses by exposure. In sub-analyses subjects with missing or incomplete outcomes were discounted for that particular measure only in year two (either depression, anxiety or PTSD); therefore the total number of participants included in denominator varies for each outcome. In the matched analyses this issue was handled at the design stage, restricting analysis to individuals with complete data in both years.

Data were entered using Epidata (Epidata Association, Denmark). The online questionnaire was designed using SelectSurvey (ClassApps, USA). Analyses were performed using Stata 12 (Statacorp, USA).

## Results

Of the 1408 participants who had consented to follow up, 1064 responded (76%). Thirty eight exclusions were made (20 duplicates and 18 participants who reported being affected by new episodes of flooding between year one and year two) and a further 38 (4%) were excluded as those respondents did not provide sufficient information to be assigned an exposure category. Of the 988 included in the analysis, 137 participants (13%) were classified as unaffected, 512 (50%) as disrupted and 339 (33%) as flooded.

Overall, approximately 6% of the participants reported symptoms indicative of depression, 8% of anxiety and 13% of PTSD with the prevalence of all adverse mental health outcomes being higher in the flooded group than among those who were unaffected (Table 1). There were no respondents unaffected by flooding who screened positive for PTSD.

**Table 1:** Crude prevalence of mental health outcomes in year two by exposure group

Outcome	Overall cohort	Exposure group		
		Unaffected	Disrupted	Flooded
Depression	59/988 (6.0%)	2/137 (1.5%)	21/512 (4.1%)	36/339 (10.6%)
Anxiety	83/988 (8.4%)	4/137 (2.9%)	33/512 (6.4%)	46/339 (13.6%)
PTSD	129/988 (13.1%)	0/137 (0.0%)	46/512 (8.9%)	83/339 (24.5%)

The adjusted odds ratios of probable depression and anxiety were significantly elevated at 8.7 (1.9–39.8) and 5.2 (1.7–16.3) for flooded participants compared with those unaffected (Table 2). Participants disrupted by flooding had approximately two times higher adjusted odds of depression 2.5 (0.5–12.0) and anxiety 1.83 (0.6–5.6) than unaffected participants; however this result was not statistically significant at the 5% level.

**Table 2:** Crude and adjusted odds ratios (aOR) of mental health outcomes by exposure group (separated by whether subjects reported damage persisting until year two)

Outcome by exposure	Prevalence %	Crude OR (95%CI)	aOR* (95% CI)	*p* value**
Probable Depression				
Unaffected	1.5	ref	ref	
All Disrupted	4.1	2.9 (0.7;12.5)	2.5 (0.5;12.0)	
No persistent damage	3.9	2.8 (0.6;12.0)	2.3 (0.5;10.9)	0.11
Persistent damage	7.3	5.1 (0.8;31.6)	7.7 (1.0;58.1)	
All Flooded	10.6	8.3 (2.0;34.8)	8.7 (1.9;39.8)	
No persistent damage	7.2	5.4 (1.2;23.7)	5.8 (1.2;26.9)	0.04
Persistent damage	20.5	17.1 (3.8;77.0)	14.0 (2.9;67.9)	
Probable Anxiety				
Unaffected	2.9	ref	ref	
All Disrupted	6.4	2.4 (0.8;6.8)	1.8 (0.6;5.6)	
No persistent damage	5.4	2.0 (0.7;5.7)	1.4 (0.4;4.4)	0.001
Persistent damage	19.5	7.9 (2.2;27.9)	9.2 (2.3;37.1)	
All Flooded	13.6	5.5 (1.9;15.6)	5.2 (1.7;16.3)	
No persistent damage	10.8	4.2 (1.4;12.4)	4.0 (1.2;12.8)	0.07
Persistent damage	21.8	9.4 (3.0;29.3)	8.4 (2.4;29.3)	
Probable PTSD				
Unaffected	0	ref	ref	
All Disrupted	8.9	∞ (3.7;∞)	not estimable	
No persistent damage	6.9	ref2***	ref2***	0.000
Persistent damage	34.1	6.8 (3.2;14.4)	10.7 (4.4;26.2)	
All Flooded	24.5	∞ (12.7;∞)	not estimable	
No persistent damage	18.4	Ref2***	Ref2***	0.000
Persistent damage	43.6	3.7 (2.1;6.6)	5.2 (2.6;10.3)	

Note: total participants *n* = 988; total participants with persistent damage *n* = 119; total participants without persistent damage *n* = 846; missing *n* = 23

*Adjusted odds ratios are adjusted for age, sex, pre;existing illness, deprivation, local authority, ethnicity, marital, education and employment statuses

**the *p*;values are for the test of the null hypothesis that the aORs for those with and without persistent damage are equal

***for PTSD, because there were no cases in the unaffected group, the group without persistent damage was taken as the reference group when comparing this group with that with persistent damage

The odds ratios of probable PTSD by exposure group were not quantifiable as no cases were reported in the reference (unaffected) group, but the substantial excess in disrupted and flooded groups could be shown to be significant in the unadjusted comparison at least, as indicated by lower confidence limits above 1 (Table 2).

Past exposure to flooding, housing tenure, insurance status at winter 2013/14, status of insurance claim and sole adult occupancy did not significantly modify the association between flooding and prevalence of psychological morbidity.

In year two, 119 participants (14%) reported persistent damage to their home as a consequence of the original episode of flooding or the disruption caused by flooding; of these 41 (34%) were classified as disrupted and 78 (65%) as flooded. Among those who reported persistent damage, the most commonly reported concerns were related to damp (8.2%) or visible mould in liveable rooms (5.6%), problems with sewage or drains (4.2%), and problems with other utilities. Sixteen participants reported that they have not yet regained the use of all liveable rooms in their home.

Even in participants without persistent damage to their home at year two, the prevalence of mental health outcomes was significantly higher in flooded participants than unaffected. Persistent damage (yes/no) was considered as sub-groups of the flooded and disrupted exposure categories; we looked at the differences in mental health outcomes between those with and without persistent damage in year two (Table 2). The odds of probable psychological morbidity amongst flooded participants who reported persistent damage was higher than amongst flooded participants who did not report persistent damage. As these sub-groups were each compared with those who were unaffected, there were different probabilities for having psychological morbidity. After adjustment for a priori confounders, this difference was significant for depression and PTSD (*p* = 0.04 and *p* < 0.001), but only suggestively so for anxiety (*p* = 0.07). Among the disrupted group, the persistent damage was a significant factor for increased adjusted odds of anxiety and PTSD (*p* = 0.001 and *p* < 0.001) but not convincingly for depression (*p* = 0.11) (Table 2).

We compared the prevalence of probable psychological morbidity between year one and year two.

We observed a significant reduction in prevalence across all mental health outcomes, in all exposure groups (Table 3).

**Table 3:** Change in prevalence of mental health outcomes from year one to year two by exposure group (matched analysis)

Outcome	Total number of respondents	Number of participants year one	Prevalence year one (%)	Number of participant year two	Prevalence year two (%)	Reduction (%)	95% Confidence interval
Depression	873	92	10.5	59	6.8	3.8	(1.5;6.1)
Flooded	294	56	19.1	36	12.2	6.8	(1.4;12.2)
Disrupted	457	32	7.0	21	4.6	2.4	(0.3;5.1)
Unaffected	122	4	3.3	2	1.6	1.6	(2.4;5.7)
Anxiety	870	149	17.1	83	9.5	7.6	(4.6;9.9)
Flooded	294	90	30.6	46	15.7	15.0	(9.2;20.7)
Disrupted	452	48	10.6	33	7.3	3.3	(0.1;6.5)
Unaffected	124	8	6.5	4	3.2	3.2	(2.5;9.0)
PTSD	896	186	20.8	127	14.2	6.6	(3.9;9.2)
Flooded	300	111	37.0	81	27.0	10.0	(4.5;15.5)
Disrupted	464	67	14.4	46	9.9	4.5	(1.0;8.1)
Unaffected	132	8	6.1	0	0.0	6.1	(1.2;10.9)

In the flooded group the reduction was greatest for anxiety, followed by PTSD and depression, however in the disrupted group the reduction was greatest for PTSD, followed by anxiety and depression. Those unaffected by flooding also showed large reductions for all mental health outcomes.

## Discussion

Living through a flood event can be distressing. The consequence for people’s mental health can be profound. After following up the health status of our participants two years after event, the observed crude prevalence of mental health outcomes was highest for PTSD, followed by anxiety and depression. Our findings demonstrate that the prevalence of probable depression, anxiety and PTSD remain elevated two years after exposure to flooding, indicating that floods have a negative impact on probable mental health outcomes for an extended period of time for people whose homes were flooded. The overall pattern of association of psychological morbidity with flooding that was observed in year one continued also in year two; however the association with disruption, though present, was no longer statistically significant.

In this study, analysis of paired observations for individuals revealed changes in mental health outcomes between year one and year two. Although the prevalence of probable psychological morbidity remains elevated two years after flooding, it declined significantly between the first and the second years of follow up. None of the variables considered a priori as putative predictors of the year one to year two reduction in psychological morbidity (flood related factors, disruption related factors and demographic variables) were found to be significant at the 5% level. This implies, in particular, that odds ratios of outcomes by exposure to flooding did not change between years more than could be explained by chance.

We also observed a (smaller) reduction in probable psychological morbidity among those unaffected by flooding; no definite information obtainable to explain these results. It is possible that some of the people classified as unaffected may have nonetheless experienced distress arising from flooding affecting their local community, as a result of disruption that was not assessed in our questionnaire or as a result of heightened risk perception relating to future flooding.

Our finding of persistent elevation in levels of psychological morbidity beyond 12 months is in keeping with other cross-sectional research after natural disasters [21]. However, we have added insight on the rate of change from 12 to 24 months in populations both directly and indirectly affected by flooding. In year one the prevalence of probable depression amongst flooded participants (*n* = 622) was 20.1%, anxiety (28.3%) and PTSD (36.2%) and amongst disrupted participants (*n* = 1099) the prevalence of probable depression was 9.6%, anxiety (10.7%) and PTSD (15.2%). In year two, the prevalence of probable psychological morbidity remained elevated amongst flooded participants [*n* = 339] (depression 10.6%, anxiety 13.6%, PTSD 24.5%) and disrupted participants [*n* = 512] (depression 4.1%, anxiety 6.4%, PTSD 8.9%). The increased prevalence we describe is in keeping with recent research quantifying community level increases in medical prescribing for common mental health disorders after floods in England [22]. Further assessment of help-seeking behaviour following flooding is needed.

This study also identified that 14% of people experienced persistent damage to their home as a consequence of the flooding or flood-related disruption in winter 2013/14, including damp, visible mould in liveable rooms, sewage-related issues or problems with other utilities. Participants who experienced persistent damage to their homes as a result of flooding had greater odds of psychological morbidity compared to those who reported no persistent damage. Merdjanoff also characterised the different impact of levels of housing damage on distress amongst displaced individuals after hurricane Katrina [23]. A better understanding of the impact of different types of damage – short or long term –experienced by individuals would be useful in helping to prevent psychological morbidity; in our study participants reported a range of damage to their homes, gardens, property and personal possessions [23].

Despite the availability of information and advice, the recovery period may be prolonged for some affected persons [24]. Our study adds to the evidence that potentially traumatic events such as floods may negatively affect survivors for a long period of time and, furthermore, the disturbance to people’s lives does not end when the flood water recedes [11, 13, 25]. Our research suggests that support to deal with the extended damage to homes, sanitation and utilities caused by flooding might be needed to reduce mental health risks. Previous research conducted as part of this cohort study has revealed that the impact of flooding on mental health can be further exacerbated due to secondary stressors (such as reporting concerns about health and the loss of items of sentimental value) [26]. By strictly looking at those individuals whose homes were flooded, Munro reported that receiving warnings had a protective effect in terms of psychological morbidity [27]. This is important, as mental health resilience could be significantly improved by providing the population with adequate information [14].

### Strengths and limitations

This study provides one of the few existing assessments of the mental health of populations exposed to flooding and disruption from flooding within local geographical areas. To our knowledge, this is the first study to have examined the impact of flooding on probable mental health outcomes two years after exposure. The longitudinal design has allowed us effectively quantify the prevalence and duration of probable psychological morbidity in people affected by flooding. Our approach to use paired comparisons for exploration of changes in prevalence, preventing confounding by changes in sociodemographic factors between year one and two. In addition, conditional logistic regression analyses allowed us to check for the modification of change in odds of outcomes by factors including exposure group. Our study may not be representative of all populations affected by flooding, as the areas affected by flooding in the winter of 2013/14 included in this study were relatively affluent areas with older populations and a large percentage of home owners. However, the unaffected comparison group came from the same areas, and factors such as age, sex, pre-existing illness, deprivation, local authority, ethnicity, marital, education and employment statuses were adjusted for as potential confounders. We found no significant predictors of the change in psychological morbidity between years one and two that could explain which groups were most likely to recover or to have persistent morbidity. This is may be due to lack of power to detect such relatively complex patterns.

Longitudinal studies are also subject to losses to follow up. The small size and low prevalence of probable mental health outcomes in the unaffected group resulted in significant uncertainty around the estimated odds ratios of psychological morbidity in the flooded and disrupted groups (Table 1).

Like most surveys, this study will have been affected by non-response [28]. Subjects in the target population were omitted if they did not respond in year one, did not consent to a further survey, or consented but did not respond to the year two survey. All these factors have then potential to bias the associations between year two prevalence and flooding (Table 2), but would only have done so if response was differential with respect to both flooding and adverse mental health. Such bias is possible, but not obviously likely to be substantial. In addition, our results on changes in prevalence between years one and two (Table 3) were based on people responding in both surveys, so relatively robust to non-response.

## Conclusions

This study has identified that the adverse impact of flooding and disruption from flooding on probable mental health persists for at least two years after exposure, however the prevalence of probable psychological morbidity reduced in the period between one year and two years after flooding. People who reported persistent flood related damage in their homes had higher odds of probable psychological morbidity. There are likely to be significant health gains from repairing properties as soon as possible and from increasing access to effective psychological services. Commissioners and providers of health and social care services should be aware of an increased need in populations affected by flooding for a prolonged period of time, at least up to two years after the event.

This study expands the knowledge about the health impact of flooding and will help inform the work of those with responsibilities to plan and respond to such events to reduce the burden of psychological morbidity. These findings should be used to inform planning for future flooding events, to strengthen multi-agency emergency response and recovery plans taking into account not just the needs of those whose homes are flooded but also those whose lives are disrupted by flooding. By applying the existing knowledge of the mental health impacts of flooding, risk reduction strategies and flood relief schemes can be readily influenced to meet flood survivors’ immediate and long-term needs.

## Additional file

Additional file 1: Survey Questionnaire. (ODT 168 kb)

## Abbreviations

aOR: Adjusted Odds Ratio
IMD: Index of Multiple Deprivation
PTSD: Post Traumatic Stress Disorder
UK: United Kingdom
WHO: World Health Organization
