# Supplementary material for: The English National Cohort Study of Flooding and Health: the change in the prevalence of psychological morbidity at year two
Source: BMC Public Health. 2018 Mar 7;18:330. doi: 10.1186/s12889-018-5236-9 (PMC5842606; doi:10.1186/s12889-018-5236-9)
Supplement: Supplementary file 1 [file 12889_2018_5236_MOESM1_ESM.odt]

National study on flooding and health: Year 2

King’s College London Research Ethics Committee Ref: PNM/13/14-152

| Thank you for completing this questionnaire, the results of which will be very important to local authorities and health services in planning and improving NHS services for communities across England. |
| --- |

Thank you for completing the first flooding questionnaire we sent you last year regarding your experience of the storms and floods that took place in the winter of 2013/14.  This follow up questionnaire captures what has happened since then.

We are carrying out this study because we believe that flooding is an important public health issue and we want to better understand the long term impacts of flooding on health.

The results will be very important to local authorities and health services in planning and improving NHS services for communities and individuals across England. This information will help us to describe the particular types of disruptions and exposures caused by flooding to quantify the medium and long-term health impact.

Part 1:Your home 

- Have you moved to a new home since we first wrote to you? (please tick one statement that applies to you)		

- No, I live in the same home ☐

- Yes, I have moved to a new home permanently with no intentions to move again in near future☐ 

Current postcode: _________

- Yes, I have moved to a new home temporarily and intend to move again in near future  	☐ 

Current postcode: __________

- Have you been affected by new episodes of flooding since we first wrote to you (approximately a year ago)? Please tick one statement which applies to you. If yes, please write the exact date if possible, otherwise provide your best guess.

|  |  |  | ( please give date: DD/MM/YYYY) |  |  |
| --- | --- | --- | --- | --- | --- |
| Yes, my home has flooded again (water entering the house) |  | ☐ | ____________ | Stop here! Please return the questionnaire in the enclosed envelope. We will send you another more appropriate questionnaire about your experience of flooding. |  |
| Yes, my home utilities were disrupted again due to flooding (e.g. electricity, gas, drinking water, sewerage, oil delivery/supply, landline, internet) |  | ☐ | DD/MM/YYYY __________ |  Stop here! Please return the questionnaire in the enclosed envelope. We will send you another more appropriate questionnaire about your experience of flooding. |  |
| Yes, my neighbourhood has been disrupted again due to flooding (e.g. access to work, shops, health care, social care, schools, education, public transport) |  | ☐ | DD/MM/YYYY __________ |  Continue to Question 3 |  |
| No, I have not been affected by new episodes of flooding. |  | ☐ |  | Continue to Question 3 |  |

- Is your home affected by any ongoing flood-related problems, caused by the flooding in 2013/14? (please tick one)

A. No not affected   ☐  Go to Question 7

B. Yes, continued flood-related problems ☐

- Please, can you specify which problems have continued since we first wrote to you?

* ‘Liveable rooms’ are your living room, kitchen, bedroom or dining room (that is, any room you occupy for at least three months of the year but not rooms such as the hallway, garage or garden). (Please tick all that apply)

A. Problems with damp in liveable rooms   ☐    

B. Visible mould in liveable rooms  ☐	            

C. Problems with damp or water in non-liveable rooms (garage, cellar or basement)   ☐ 

D. Sewage (drains) backing up and flooding  ☐

E. Problems with septic tank ☐

F. Problems with other utilities (drinking water, gas, oil, electricity, etc.) ☐

G. Other   ☐   Please specify what other problems have continued: _____________________________________

_________________________________________________________________________________

_________________________________________________________________________________

_________________________________________________________________________________

_________________________________________________________________________________

- Since the storms and flooding in winter 2013/2014, if your home was flooded, have you been able to resume use of all liveable rooms in the property as normal? (please tick one)

A. Yes, I have been able to resume using all the liveable rooms as normal		☐

B. No, I have not yet been able to resume using all the liveable rooms as normal	☐

C. Not applicable ☐

- How much of the following payments, if any, have now been approved/paid to you relating to winter 2013/2014 flood damage in your home? (please tick one in each column)

|  | Repair and renew grant application | Home and household insurance claims |
| --- | --- | --- |
| All money has been paid and there is none outstanding | ☐ | ☐ |
| Some money has been paid; there is still some outstanding | ☐ | ☐ |
| None of the money has been paid | ☐ | ☐ |
| Not applicable | ☐ | ☐ |

7. Please, answer the following questions about your experience, since the first time we wrote to you. Please answer the first and the second column even if you were not flooded. (Please answer each row). 

|  | Which of the following did you experience, since we first wrote to you (approx. a year ago) | If yes, how stressful was this for you? | Did you experience this because of flooding in/near your home? |
| --- | --- | --- | --- |
| Yes | No | N/A | Mild/No stress | Moderate stress | Severe stress | Yes | No |
| Dealing with insurance issues | ☐ | ☐ | ☐ | ☐ | ☐ | ☐ | ☐ | ☐ |
| Repairing my home | ☐ | ☐ | ☐ | ☐ | ☐ | ☐ | ☐ | ☐ |
| Living in a damaged home | ☐ | ☐ | ☐ | ☐ | ☐ | ☐ | ☐ | ☐ |
| Concerns about my health | ☐ | ☐ | ☐ | ☐ | ☐ | ☐ | ☐ | ☐ |
| Concerns about my family’s health | ☐ | ☐ | ☐ | ☐ | ☐ | ☐ | ☐ | ☐ |
| Relationship problems (e.g. arguing with partner/friends/children) | ☐ | ☐ | ☐ | ☐ | ☐ | ☐ | ☐ | ☐ |
| Spending more time helping family, friends or community members | ☐ | ☐ | ☐ | ☐ | ☐ | ☐ | ☐ | ☐ |
| Disagreements/arguments with neighbours | ☐ | ☐ | ☐ | ☐ | ☐ | ☐ | ☐ | ☐ |
| Concerns about the value of your home | ☐ | ☐ | ☐ | ☐ | ☐ | ☐ | ☐ | ☐ |

Part 2 - Your wellbeing

These questions are used to describe your wellbeing. Please answer these questions even if you or your home were not affected by flooding, so that we can make comparisons between different groups of people.

- Under each heading, please tick the ONE box that best describes your health TODAY[^1]. 

| MOBILITY |  |
| --- | --- |
| I have no problems in walking about |  |
| I have slight problems in walking about |  |
| I have moderate problems in walking about |  |
| I have severe problems in walking about |  |
| I am unable to walk about |  |
| SELF-CARE |  |
| I have no problems washing or dressing myself |  |
| I have slight problems washing or dressing myself |  |
| I have moderate problems washing or dressing myself |  |
| I have severe problems washing or dressing myself |  |
| I am unable to wash or dress myself |  |
| USUAL ACTIVITIES (e.g. work, study, housework, family or leisure activities) |  |
| I have no problems doing my usual activities |  |
| I have slight problems doing my usual activities |  |
| I have moderate problems doing my usual activities |  |
| I have severe problems doing my usual activities |  |
| I am unable to do my usual activities |  |
| PAIN / DISCOMFORT |  |
| I have no pain or discomfort |  |
| I have slight pain or discomfort |  |
| I have moderate pain or discomfort |  |
| I have severe pain or discomfort |  |
| I have extreme pain or discomfort |  |

| ANXIETY / DEPRESSION |  |
| --- | --- |
| I am not anxious or depressed |  |
| I am slightly anxious or depressed |  |
| I am moderately anxious or depressed |  |
| I am severely anxious or depressed |  |
| I am extremely anxious or depressed |  |

| We would like to know how good or bad your health is TODAY |
| --- |
| This scale is numbered from 0 to 100. |
| 100 means the best health you can imagine. 0 means the worst health you can imagine. |

| Mark an X on the scale to indicate how your health is TODAY. |
| --- |
| Now, please write the number you marked on the scale in the box below. YOUR HEALTH TODAY = Thinking over the last two weeks, how often have you been bothered by the following problems? (please tick) Not at all Several days More than half the days Nearly every day Feeling nervous, anxious or on edge ☐ ☐ ☐ ☐ Not being able to stop or control worrying ☐ ☐ ☐ ☐ Feeling down, depressed, or hopeless ☐ ☐ ☐ ☐ Little interest or pleasure in doing things ☐ ☐ ☐ ☐ |

- Below is a list of problems and complaints that people sometimes have in response to stressful experiences. Please read each one carefully and put a tick in the box to indicate how much you have been bothered by that problem in the past month. 

|  | Not at all | A little    bit | Moderately | Quite a bit | Extremely |
| --- | --- | --- | --- | --- | --- |
| Repeated, disturbing memories, thoughts, or images of a stressful experience? | ☐ | ☐ | ☐ | ☐ | ☐ |
| Feeling very upset when something reminded you of a stressful experience? | ☐ | ☐ | ☐ | ☐ | ☐ |
| Avoiding activities or situations because they reminded you of a stressful experience? | ☐ | ☐ | ☐ | ☐ | ☐ |
| Feeling distant or cut off from other people? | ☐ | ☐ | ☐ | ☐ | ☐ |
| Feeling irritable or having angry outbursts? | ☐ | ☐ | ☐ | ☐ | ☐ |
| Having difficulty concentrating? | ☐ | ☐ | ☐ | ☐ | ☐ |

- If you checked off any problems in the previous question, how difficult have these problems made it for you to do your work, take care of things at home, or get along with other people? (please tick)

Not at all difficult	☐ 			Somewhat difficult   ☐	N/A   ☐

Very difficult        ☐			Extremely difficult   ☐

- During the past 4 weeks, how much have you been bothered by any of the following problems? (please tick)

|  | Not bothered at all | Bothered a little | Bothered a lot |
| --- | --- | --- | --- |
| Stomach pain | ☐ | ☐ | ☐ |
| Back pain | ☐ | ☐ | ☐ |
| Pain in your arms, legs, or joints (knees, hips, etc.) | ☐ | ☐ | ☐ |
| Headaches | ☐ | ☐ | ☐ |
| Chest pain | ☐ | ☐ | ☐ |
| Dizziness | ☐ | ☐ | ☐ |
| Fainting spells | ☐ | ☐ | ☐ |
| Feeling your heart pound or race | ☐ | ☐ | ☐ |
| Shortness of breath | ☐ | ☐ | ☐ |
| Pain or problems during sexual intercourse | ☐ | ☐ | ☐ |
| Constipation, loose bowels, or diarrhoea | ☐ | ☐ | ☐ |
| Nausea, wind, or indigestion | ☐ | ☐ | ☐ |
| Feeling tired or having low energy | ☐ | ☐ | ☐ |
| Trouble sleeping | ☐ | ☐ | ☐ |
| Women only: Menstrual cramps or other problems with your periods | ☐ | ☐ | ☐ |

- Have you sought help or treatment for any stress-related, mental or emotional problem since the 2013/14 flooding? Seeking help means any attempt to get advice, support or treatment from any of the groups listed below: (please tick one in each row)

| No Yes, in the past four weeks Yes, in the past year Local GP ☐ ☐ ☐ Practice nurse ☐ ☐ ☐ Mental health services ☐ ☐ ☐ Another health professional ☐ ☐ ☐ Voluntary group / charity                                                       ☐ ☐ ☐ Family / friends                          ☐ ☐ ☐ Other, please specify ___________________________________________ |  |  |  |  |  |  |  |
| --- | --- | --- | --- | --- | --- | --- | --- |

- Have you received treatment (including medication or other forms of treatment, such as counselling) for any stress-related, mental or emotional problem since the 2013/14 flooding from any of the groups listed below? (please tick one in each row)

|  | No | Yes, in the past four weeks | Yes, in the past year |
| --- | --- | --- | --- |
| Local GP | ☐ | ☐ | ☐ |
| Practice nurse | ☐ | ☐ | ☐ |
| Mental health services | ☐ | ☐ | ☐ |
| Another health professional | ☐ | ☐ | ☐ |
| Voluntary group/charity | ☐ | ☐ | ☐ |
| Other, please specify |  |  |  |
| ___________________________________________ |  |  |  |
|  |  |  |  |

- Overall, the amount of help and support I received for non-health related issues arising from flooding, from the groups below, was : (Please tick one statement that applies to you in each column)

|  | Family/friends/neighbours | Local council/official agencies | Voluntary groups |
| --- | --- | --- | --- |
| Much less than I needed | ☐ | ☐ | ☐ |
| Less than I needed | ☐ | ☐ | ☐ |
| About right | ☐ | ☐ | ☐ |
| More than I needed | ☐ | ☐ | ☐ |
| Much more than I needed | ☐ | ☐ | ☐ |
| Not applicable | ☐ | ☐ | ☐ |

PART 3 – Questions about you and where you live

These questions will help us to know that we have a good cross-section of the community taking part in the study. Some of these factors can affect health and wellbeing.

- Which of these best describes your marital status? (please tick one)

Single, never married or never in a civil partnership   ☐

Married or in a civil partnership   ☐          Separated  ☐                                 

Widowed  ☐                Divorced  ☐        Cohabiting  ☐

Other        ☐   Please specify: ________________________________________________

- Which of the following best describes your education level? (please tick one)

Degree or equivalent   ☐				Below degree level   ☐

Other (foreign or other qualification)   ☐	None (no formal qualification)   ☐

- Which of the following best describes your employment status? (please tick one) 

| Working full time (30hrs/wk+) | ☐ |
| --- | --- |
| Working part time (8–29 hrs/wk) | ☐ |
| Stay-at-home parent / Homemaker / Housewife | ☐ |
| Unemployed (registered) | ☐ |
| Unemployed (not registered but looking for work) | ☐ |
| Retired | ☐ |
| Student | ☐ |
| Other (inc. disabled) | ☐ |
| Don’t know | ☐ |
| Would prefer not to say | ☐ |

 

- Currently, do you have any long-term illness, health problem or disability which limits your daily activities or the work you can do? (please tick)

Yes     ☐		No     ☐

If yes, please tell us what (eg depression, diabetes etc): __________________________________________

__________________________________________

__________________________________________

- We would like to contact you again next year to help with this study or any other queries we might have, please provide us with your contact details.

Address where I can be reached

Name:     __________________________________________

First Line:     __________________________________________

Second Line: __________________________________________

Third Line:    __________________________________________

Postcode:       __________________________________________

E-mail:         __________________________________________

Telephone or Mobile phone:    ___________________________

Please, can you indicate how you would like us to contact you in future:

By post including paper questionnaires				☐

Email, including details of online questionnaires			☐

I would like to receive an electronic summary of the results via email	☐	

If you have any comments, please write them below:

___________________________________________________________________________________

___________________________________________________________________________________

___________________________________________________________________________________

Thank you for your participation

[^1]: © EuroQol Research Foundation. EQ-5D™ is a trade mark of the EuroQol Research Foundation.
